# Influenza Vaccination Coverage Among Pregnant Women — United States, 2013–14 Influenza Season

**Published:** 2014-09-19

**Authors:** Helen Ding, Carla L. Black, Sarah Ball, Sara Donahue, David Izrael, Walter W. Williams, Erin D. Kennedy, Carolyn B. Bridges, Peng-Jun Lu, Katherine E. Kahn, Lisa A. Grohskopf, Indu B. Ahluwalia, John Sokolowski, Charles DiSogra, Deborah K. Walker, Stacie M. Greby

**Affiliations:** 1 Immunization Services Division, National Center for Immunization and Respiratory Diseases, CDC; 2 Abt Associates, Inc., Cambridge, Massachusetts; 3 Influenza Division, National Center for Immunization and Respiratory Diseases, CDC; 4 Division of Reproductive Health, National Center for Chronic Disease Prevention and Health Promotion, CDC; 5 Abt SRBI, New York, New York

Pregnant women and infants are at increased risk for influenza-related complications and hospitalization. Influenza vaccination among pregnant women can reduce their risk for respiratory illness and reduce the risk for influenza in their infants aged <6 months (1). Since 2004, the Advisory Committee on Immunization Practices and the American College of Obstetricians and Gynecologists have recommended influenza vaccination for all women who are or will be pregnant during the influenza season, regardless of trimester (1,2). To assess influenza vaccination coverage among pregnant women during the 2013–14 influenza season, CDC analyzed data from an Internet panel survey conducted March 31–April 11, 2014. Among 1,619 survey respondents pregnant at any time during October 2013–January 2014, 52.2% reported vaccination before or during pregnancy (17.6% before and 34.6% during pregnancy), similar to the coverage in the preceding season. Overall, 65.1% of women reported receiving a clinician recommendation and offer of influenza vaccination, 15.1% received a clinician recommendation but no offer of vaccination, and 19.8% received no clinician recommendation or offer. Vaccination coverage among these women was 70.5%, 32.0%, and 9.7%, respectively. Continued efforts are needed to encourage clinicians to strongly recommend and offer influenza vaccination to their pregnant patients.

An Internet panel survey was conducted for CDC by Abt Associates, Inc. (Cambridge, Massachusetts) during March 31–April 11, 2014, to 1) provide end-of-season estimates of influenza vaccination coverage among pregnant women for the 2013–14 influenza season; 2) assess clinician recommendation and offer of influenza vaccination; and 3) obtain updated information on women’s knowledge, attitudes, and behaviors related to influenza vaccination. Women aged 18–49 years who reported being pregnant at any time after August 2013 were eligible for the survey. Participants were recruited from a preexisting, national, opt-in, general population Internet panel operated by Survey Sampling International, which provides panel members with online survey opportunities in exchange for nominal incentives.* Pregnant women panelists were recruited from the Survey Sampling International panel using two methods: 1) an email invitation was sent to panel members aged 18–49 years, female, and living in the United States or 2) a message on the panel website inviting panel members to answer a series of screening questions and, if eligible, to take the survey. Of 12,068 women who entered the survey site, 2,127 were determined to be eligible, and 2,042 (96.0%) completed the survey.† Data were weighted to reflect the age, race/ethnicity, and geographic distribution of the total U.S. population of pregnant women. A woman was considered to be vaccinated if 1) vaccination was received during July 2013–April 2014, and 2) vaccination was received before or during the most recent pregnancy. The study population was limited to women who reported pregnancy any time during the peak influenza vaccination period of October 2013–January 2014 (N = 1,619). Vaccination coverage estimates from the Internet panel surveys completed for the 2010–11 through 2013–14 seasons were compared to assess trends over time. Similar methodology was used in all four survey years (3).

Survey respondents were asked questions about their vaccination status before and during pregnancy, whether their clinician recommended and offered influenza vaccination, their attitudes regarding influenza and influenza vaccination, and their reasons for receiving or not receiving influenza vaccination. Three composite variables defining attitudes toward influenza vaccination efficacy, influenza vaccination safety, and concerns about influenza infection were constructed using methods previously described (3). Because the opt-in Internet panel sample is not probability-based, no statistical tests were performed.§ Differences were noted when there was a difference of ≥5 percentage points between any values being compared.

Of the 1,619 women pregnant at any time during October 2013–January 2014, 52.2% reported receiving influenza vaccination after July 1, 2013 (17.6% before and 34.6% during pregnancy). The overall vaccination coverage was similar to coverage in the 2012–13 influenza season (50.5%) but higher than that in the 2011–12 (46.4%) and 2010–11 seasons (44.0%) (Figure). Non-Hispanic black women had the lowest vaccination coverage (42.7%) compared with women of the other three racial/ethnic groups (non-Hispanic white: 52.0%, Hispanic: 56.7%, and non-Hispanic, other race: 61.9%). Women with the following reported characteristics had lower influenza vaccination coverage than other women within each comparison stratum: those aged 18–24 years, with education less than a college degree, not married, reporting no medical insurance, not working for wages, living below the poverty threshold, reporting no high-risk conditions associated with increased complications for influenza, reporting fewer than six visits to a clinician since July 2013, and having a negative attitude towards efficacy and safety of influenza vaccination or not being concerned about influenza infection. Vaccination coverage increased from 2012–13 to 2013–14 for Hispanic women, non-Hispanic women who reported race other than white or black, women aged 25–34 years, and women with greater than a college degree (Table 1).

What is already known on this topic?

Pregnant women and infants are at increased risk for influenza-related complications and hospitalization. Influenza vaccination among pregnant women can reduce their risk for respiratory illness and reduce the risk for influenza in their infants aged <6 months. Influenza vaccination coverage among pregnant women increased substantially during the 2009–10 influenza season, and the increased coverage was sustained during the 2010–11 through 2012–13 seasons.

What is added by this report?

In the 2013–14 influenza season, 52.2% of pregnant women were vaccinated before or during pregnancy; 65.1% of women reported receiving a clinician recommendation and offer of influenza vaccination, an increase of about 10 percentage points from the 2012–13 season. Women who received a clinician offer of vaccination had higher vaccination coverage than those who did not receive an offer of vaccination. Barriers to vaccination included negative attitudes toward safety and efficacy of influenza vaccination and perceptions of low personal risk for influenza.

What are the implications for public health practice?

Continued efforts are needed to increase knowledge among pregnant women about the safety and efficacy of influenza vaccination and the risk for influenza for themselves and their infants. Additionally, efforts are needed to increase opportunities for clinicians to recommend and offer influenza vaccination to pregnant women.

Among women with at least one visit to a clinician since July, increases were observed between the 2010–11 to 2013–14 seasons in the percentage of women who reported receiving a clinician recommendation and offer of vaccination (from 56.9% to 65.1%) (Figure). In the 2013–14 season, women who reported receiving both a clinician recommendation and offer of influenza vaccination had higher vaccination coverage (70.5%) compared with women who reported receiving a clinician recommendation but no offer (32.0%) and women who reported receiving no recommendation (9.7%). Among women who reported negative attitudes toward influenza vaccination efficacy, vaccination safety, or no concern about influenza infection but reported receiving a clinician recommendation and offer of vaccination, vaccination coverage was 15.4%, 26.1%, and 56.7%, respectively, higher than coverage among women with the same attitude but who reported only receiving a clinician recommendation (0.0%, 3.3%, and 27.6%, respectively) or receiving no recommendation (0.0%, 3.9%, and 7.9%, respectively) (Table 2).

The most common reasons women reported for receiving vaccination were to protect their infant from influenza (31.1%), to protect themselves from influenza (23.3%), and because their clinician recommended the influenza vaccination (14.8%). The most common reasons women reported for not receiving vaccination were concern the vaccination would give them influenza (16.8%), concern about possible safety risk to their infants if they got vaccinated (14.4%), and belief that they did not need the vaccination (12.2%).

## Discussion

During the 2013–14 influenza season, influenza vaccination among pregnant women was 52.2%, similar to coverage in the 2012–13 season (50.5%), but higher than the estimates in the 2011–12 season (46.4%) and 2010–11 season (44.0%) (3). Vaccination coverage among non-Hispanic black women was substantially lower compared with women of the other three racial/ethnic groups. This long-standing black-white disparity in vaccination coverage might be attributable to multiple factors, including weaker or less effective clinician recommendations, sociocultural norms, less awareness of vaccination recommendations, misperception of effectiveness and safety of vaccination, vaccination resistance and hesitancy, and poorer quality of clinician-patient relationships (4,5). Women who were younger (aged 18–24 years), reported having no medical insurance, had fewer than six visits to a clinician since July 2013, had less education, were not working, or lived below the poverty threshold also had lower vaccination coverage than other subgroups of women in the survey.

Women who reported receiving a clinician recommendation and offer of influenza vaccination had higher vaccination coverage compared with women who reported receiving only a recommendation but no offer or reported receiving no recommendation, even among those who reported having a negative attitude toward efficacy, safety of influenza vaccination, or no concern about influenza infection. These results are consistent with previous findings (3), and highlight the importance of a clinician offer of influenza vaccination to increase vaccination coverage among pregnant women. Previously reported clinician barriers to recommending and offering adult vaccination services include concern about lack of reimbursement for vaccination services and for the up-front cost of ordering vaccines, the high costs of storing and maintaining vaccine inventory, not having electronic health records, the inability to assess patients’ vaccination status, not perceiving responsibility as the vaccinator, and organizational challenges of vaccine administration (6,7). Systems that support clinician ability to recommend and offer vaccination to pregnant women, such as client-based education with standing orders, clinician reminder systems, and expanded access to vaccination services in multiple health care settings (e.g., pharmacies) can increase opportunities for vaccination and improve vaccination coverage.¶

Misbelief among pregnant women not receiving vaccination that vaccination would give them “the flu,” having concerns about the safety risk to their infant if they were vaccinated, and lack of awareness about their risk for influenza were the most common reasons reported for not receiving vaccination. To help change negative attitudes and misperceptions about vaccination, clinic-based client education for pregnant women should emphasize that vaccination during pregnancy is safe and can reduce influenza risk not only for pregnant women themselves but also their infants during the first 6 months of life. Such messages can be delivered through multiple channels, including prenatal care consultation, social media, and text messaging (e.g., https://text4baby.org ).

The findings in this report are subject to at least four limitations. First, vaccination was self-reported and not validated by medical record review. Second, the results were weighted to the distribution of pregnant women in the U.S. population, but the study sample did not include women without Internet access. Therefore, results might not be generalizable to all pregnant women in the United States. Third, estimates might be biased if the selection processes for entry into the Internet panel and a woman’s decision to participate in this particular survey were related to receipt of vaccination. Fourth, the composite variables computed for attitudes toward influenza vaccination and infection were not validated.

Despite these limitations, the opt-in Internet panel survey can provide timely estimates of influenza vaccination coverage and in-depth information about knowledge, attitudes, behaviors, and barriers related to influenza vaccination among pregnant women. Trends in vaccination coverage reported from the Internet panel surveys have been consistent with those reported from other less timely data sources, such as the Behavioral Risk Factor Surveillance System (8). Additionally, comparing the 2010–11 influenza season vaccination estimates from 18 states in both the Internet panel survey and the Pregnancy Risk Assessment Monitoring System, a probability sampling survey, the Internet panel survey estimate for women pregnant at any time during October 2010–January 2011 (50.2%) was similar to the estimate from the Pregnancy Risk Assessment Monitoring System for women who were pregnant in the same period (49.2%) (3).

Clinician offer of influenza vaccination was associated with higher vaccination coverage among pregnant women, even among women with negative attitudes towards vaccination. Although more women reported receiving a clinician’s recommendation and offer of influenza vaccination compared with previous seasons, efforts to enhance clinician practices are needed. Missed opportunities for vaccination can be reduced by implementing systems to ensure vaccination is recommended and offered at each visit. If a clinician cannot offer vaccination, a referral should be provided to ensure influenza vaccination before or during pregnancy. To help pregnant women understand the importance of vaccination to them, clinicians should emphasize that vaccination is safe and can decrease the risk for influenza-related illness and complications in pregnant women and their infants (9).

## Figures and Tables

**FIGURE:**
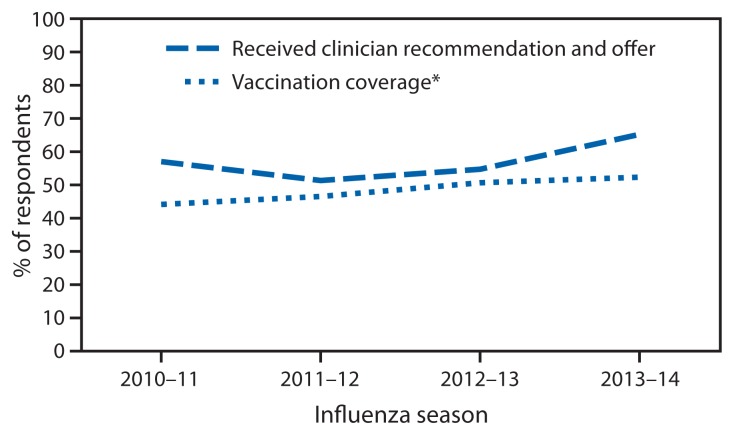
Prevalence of clinician recommendation and offer of influenza vaccination and influenza vaccination coverage before and during pregnancy among women pregnant any time during October–January — Internet panel survey, United States, 2010–11 through 2013–14 influenza seasons * Vaccination coverage estimates for the 2012–13 and 2013–14 influenza seasons were based on vaccinations given from July to mid-April. Coverage estimates for the 2010–11 and 2011–12 influenza seasons were based on vaccinations given from August to mid-April.

**TABLE 1:** Influenza vaccination coverage before and during pregnancy among women who were pregnant any time during October–January, by selected characteristics — Internet panel survey, 2013–14 and 2012–13 influenza seasons

Characteristic	2013–14 influenza season			2012–13 influenza season			Vaccination coverage difference (percentage points)
	
	Unweighted no.	Weighted %	Vaccinated weighted %	Unweighted no.	Weighted %	Vaccinated weighted %	
**Total**	**1,619**		**52.2**	**1,702**	**—**	**50.5**	**1.7**
Vaccinated before pregnancy	289		17.6	239		14.6	3.0
Vaccinated during pregnancy	577		34.6	638		35.9	−1.3
**Age group (yrs)**							
18–24	373	34.0	45.6	477	33.1	48.7	−3.1
25–34	942	50.4	56.5	970	50.5	50.5	6.0
35–49	304	15.6	53.0	255	16.3	54.1	−1.1
**Race/Ethnicity**							
Hispanic	260	23.7	56.7	278	23.8	50.1	6.6
Black, non-Hispanic	160	18.1	42.7	175	18.8	45.4	−2.8
White, non-Hispanic	1,033	50.1	52.0	1,093	50.3	52.2	−0.2
Other, non-Hispanic	166	8.1	61.9	156	7.2	53.1	8.8
**Education**							
Less than college degree	699	47.7	44.6	844	51.8	43.9	0.7
College degree	714	41.1	57.4	656	36.8	57.3	0.1
Greater than college degree	206	11.2	65.9	202	11.4	58.5	7.4
**Married**							
Yes	1,128	63.4	56.6	1,120	62.2	54.8	1.8
No	491	36.6	44.7	582	37.8	43.5	1.2
**Insurance coverage**							
Any public	579	40.0	51.0	659	41.8	50.0	1.0
Private/Military only	993	56.6	54.9	939	51.7	53.0	1.9
No insurance	47	3.3	22.2	104	6.5	33.7	−11.7
**Working status** *							
No	764	49.2	47.0	860	50.4	44.7	2.3
Yes	855	50.8	57.3	842	49.6	56.4	0.9
**Poverty status** †							
Below poverty	250	18.5	45.0	404	26.0	41.6	3.4
At or above poverty	1,369	81.5	53.9	1,289	74.0	53.8	0.1
**High-risk conditions** §							
Yes	538	33.0	60.5	613	36.3	57.8	2.8
No	1,081	67.0	48.2	1,089	63.7	46.4	1.8
**No. of visits to a clinician since July**							
No visit	16	0.9	—¶	27	1.5	—¶	
1–5 visits	370	23.2	42.7	682	41.6	48.0	−5.3
6–10 visits	652	40.4	55.0	598	34.9	53.1	1.9
>10 visits	581	35.5	56.5	395	21.9	53.1	3.3
**Clinician recommendation and/or offer** **							
Recommended and offered	1,037	65.1	70.5	895	54.6	70.5	0.0
Recommended with no offer	242	15.1	32.0	270	16.7	46.3	−14.3
No recommendation	324	19.8	9.7	455	28.7	16.1	−6.4
**Attitude toward efficacy of influenza vaccination** ††							
Negative	303	18.7	5.8	430	25.2	9.8	−4.0
Positive	1,316	81.3	62.9	1,272	74.8	64.2	−1.3
**Attitude toward safety of influenza vaccination** §§							
Negative	378	24.8	13.2	475	28.7	13.0	0.2
Positive	1,241	75.2	65.1	1,227	71.3	65.6	−0.5
**Attitude toward influenza infection** ¶¶							
Not concerned	492	30.2	39.0	564	36.9	49.9	−9.1
Concerned	1,127	69.8	58.0	939	63.1	54.1	3.9

* Those employed for wages and self-employed were grouped as working. Those who were out of work, homemakers, students, retired, or unable to work were grouped as not working.

† Below the poverty threshold was defined as a total of annual family income of <$23,283 for a family of four with two minors as of 2012, as categorized by the U.S. Census Bureau (http://www.census.gov/hhes/www/poverty/data/threshld).

§ Conditions associated with increased risk for serious medical complications from influenza, including chronic asthma, a lung condition other than asthma, a heart, kidney, or liver condition, diabetes, obesity, or a weakened immune system caused by a chronic illness or by medications taken for a chronic illness.

¶ Vaccination coverage estimates were not reliable because sample size was <30.

** Women were excluded if they did not visit a clinician after August 2013 (n = 16) for the 2013–14 influenza season, did not visit a clinician after August 2012 (n = 27), or did not know whether they received a clinician recommendation or offer (n = 55) for the 2012–13 influenza season.

†† A composite variable about attitude toward influenza vaccination efficacy was created based on two questions regarding attitudes toward influenza vaccination, “Flu vaccine is somewhat/very effective in preventing flu,” and “Agree/Strongly agree that if a pregnant woman receives the flu vaccination, it will protect the baby from getting the flu after it is born.” For the 2013–14 influenza season, the second question was slightly different: “The flu vaccine a pregnant woman receives is somewhat/very effective in protecting her baby from the flu.” One point was given for each “yes” answer for either of the two questions. Respondents with a summary score of 1 or 2 were considered as having a “positive” attitude, and those with a summary score of 0 were considered as having a “negative” attitude.

§§ A composite variable about the attitude toward influenza vaccination safety was created based on three questions regarding the safety of influenza vaccination: “Flu vaccination is somewhat/very/completely safe for most adult women,” “Flu vaccination is somewhat/very/completely safe for pregnant women,” and “Flu vaccination that a pregnant woman receives is somewhat/very/completely safe for her baby.” One point was given for each “yes” answer for any of the three questions. Respondents who had a summary score of 2 or 3 were considered as having a “positive” attitude, and those with a summary score of 0 or 1 were considered as having a “negative” attitude.

¶¶ A composite variable about the attitude toward influenza infection was created for the 2012–13 influenza season based on response to a single question regarding attitude toward influenza infection: “If a pregnant woman gets the flu, it is somewhat/very likely to harm the baby.” Respondents with a “yes” answer were considered as “concerned,” and those with a “no” answer were considered as “not concerned.” For the 2013–14 influenza season, two more questions were added: Respondent was “somewhat/very worried about getting sick with the flu this season,” and “If a pregnant woman gets the flu, it is somewhat/very likely to harm her.” One point was given for each “yes” answer for any of the three questions. Respondents who had a summary score of 2 or 3 were considered as “concerned” and those with a summary score of 0 or 1 were considered as “not concerned.”

**TABLE 2:** Percentage of women receiving a clinician recommendation/offer of influenza vaccination and influenza vaccination coverage by clinician recommendation and offer, by attitude towards influenza vaccination, among women who visited a clinician at least one time since August 2013 and who were pregnant any time during October–January — Internet panel survey, United States, 2013–14 influenza season

	Clinician recommendation/offer				Vaccination coverage					
		
		Recommended and offered	Recommended without offer	No recommendation	Recommended and offered		Recommended without offer		No recommendation	
							
Attitude	No.	Weighted %	Weighted %	Weighted %	No.	Weighted %	No.	Weighted %	No.	Weighted %
**Attitude toward efficacy of influenza vaccination** *										
Negative	295	38.7	22.9	38.4	109	15.4	66	0.0	120	0.0
Positive	1,308	71.0	13.4	15.6	928	77.2	176	44.2	204	15.1
**Attitude toward safety of influenza vaccination** †										
Negative	372	43.2	20.7	36.1	155	26.1	78	3.3	139	3.9
Positive	1,231	72.3	13.3	14.4	882	79.2	164	46.6	185	14.5
**Attitude toward influenza infection** §										
Not concerned	482	58.4	16.8	24.8	271	56.7	85	27.6	126	7.9
Concerned	1,121	68.0	14.4	17.6	766	75.5	157	34.1	198	10.8

* A composite variable about attitude toward influenza vaccination efficacy was created based on two questions regarding attitudes toward influenza vaccination, “Flu vaccine is somewhat/very effective in preventing flu,” and “The flu vaccine a pregnant woman receives is somewhat/very effective in protecting her baby from the flu.” One point was given for each “yes” answer for either of the two questions. Respondents with a summary score of 1 or 2 were considered as having a “positive” attitude, and those with a summary score of 0 were considered as having a “negative” attitude.

† A composite variable about the attitude toward influenza vaccination safety was created based on three questions regarding the safety of influenza vaccination: “Flu vaccination is somewhat/very/completely safe for most adult women,” “Flu vaccination is somewhat/very/completely safe for pregnant women,” and “Flu vaccination that a pregnant woman receives is somewhat/very/completely safe for her baby.” One point was given for each “yes” answer for any of the three questions. Respondents who had a summary score of 2 or 3 were considered as having a “positive” attitude, and those with a summary score of 0 or 1 were considered as having a “negative” attitude.

§ A composite variable about the attitude toward influenza infection was created based on response to three questions regarding attitude toward influenza infection: “If a pregnant woman gets the flu, it is somewhat/very likely to harm the baby.” Respondent was “somewhat/very worried about getting sick with the flu this season,” and “If a pregnant woman gets the flu, it is somewhat/very likely to harm her.” One point was given for each “yes” answer for any of the three questions. Respondents who had a summary score of 2 or 3 were considered as “concerned” and those with a summary score of 0 or 1 were considered as “not concerned.”

## Footnotes

* Additional information available at http://www.surveysampling.com.

† An opt-in Internet panel survey is a nonprobability sampling survey. The denominator for a response rate calculation cannot be determined because no sampling frame with a selection probability is involved at the recruitment stage. Instead, the survey completion rate is provided.

§ Additional information available at http://www.aapor.org/opt_in_surveys_and_margin_of_error1.htm.

¶ Additional information available at http://www.thecommunityguide.org/vaccines/universally/communityinterventions.html.
